# Prospects for Therapies in Osteoarthritis

**DOI:** 10.1007/s00223-020-00672-9

**Published:** 2020-02-13

**Authors:** Asim Ghouri, Philip G. Conaghan

**Affiliations:** 1 Leeds Institute of Rheumatic and Musculoskeletal Medicine, University of Leeds and NIHR Leeds Biomedical Research Centre, Leeds, UK; 2 Leeds Institute of Rheumatic and Musculoskeletal Medicine, Chapel Allerton Hospital, Chapeltown Rd, Leeds, LS7 4SA UK

**Keywords:** Osteoarthritis, Nociceptive pain, DMOAD, Cartilage, Inflammation, Synovitis

## Abstract

Osteoarthritis (OA) is a chronic, debilitating disease affecting millions of people worldwide. Management of OA involves pharmacological and non-pharmacological approaches. Conventional pharmacological treatments have limited efficacy and are associated with a number of side-effects, restricting the number of patients who can use them. New pharmacological therapies for managing OA are required and a number have been developed targeting different tissues in OA: bone and cartilage, synovium and nerves. However, there has been overall limited success. Disease-modifying osteoarthritis drugs (DMOADs) are a putative class of therapies aimed at improving OA structural pathologies and consequent symptoms. Recent DMOAD studies have demonstrated some promising therapies but also provided new considerations for future trials.

## Introduction

Osteoarthritis (OA) is a chronic, painful arthritis and a major cause of disability in affected individuals. At least 242 million people globally have hip/knee OA [1]. In addition, with an ageing population and rising risk factors such as obesity, this prevalence is growing [2]. As a result of the disability caused by OA, there is a significant cost to the global economy [3]. The pathogenesis of OA is complex with mechanical, genetic, metabolic and inflammatory pathways involved in its slowly evolving process [4]. Structure–pain relationships remain difficult to understand, and at the individual patient level, it is unclear which structures may be contributing to pain. Intra-articular candidates include bone and synovium, though it is worth noting that the amount of pain that pathologies in these tissues explain is small [5], and extra-articular features (e.g. tendinitis and bursitis) may confound associations.

OA management entails pharmacological and non-pharmacological approaches; when these fail to relieve symptoms, joint replacement surgery is considered. Non-pharmacological options (especially muscle strengthening) are often underutilised, and pharmacological options have not progressed significantly for decades: primarily paracetamol, non-steroidal anti-inflammatory drugs (NSAIDs, topical and oral) and opioids. However, the beneficial effect of paracetamol and opioids are limited, while NSAIDs and opioids are unsuitable for many patients because of their side-effects [6]. Intra-articular therapy, including corticosteroids, may also be used, although their effects tend to be short-lived.

This narrative review will update clinicians with recent developments on the use of existing pharmacological therapies and discuss novel treatments currently under an advanced stage of investigation (at least in phase II trials) for the treatment of OA. The therapies discussed have mostly been investigated for the treatment of knee OA and less commonly in hand OA. We have attempted to categorise these agents according to the tissues they target: bone and cartilage, nerves and synovium (the latter categorised as “immunomodulators”); we have included studies with both symptom and structural outcomes.

Though not systematic, this review was based on a PubMed search and review of meeting abstracts on trials reported between 2017 and 2019; both positive and negative reporting trials were included. We have excluded non-pharmacological treatments, therapies marketed as devices (e.g. hyaluronans) and nutraceuticals (e.g. glucosamine). Where relevant, older studies have been referenced to give context on the candidate therapy.

## Targeting Bone and Cartilage

### Bisphosphonates

Subchondral bone plays a role in maintaining hyaline articular cartilage integrity and its pathology is integral to the OA process [7]. A number of therapies used for osteoporosis have been explored as OA therapies, targeting altered bone turnover in the subchondral region [8, 9]. Bisphosphonates have a long history of trials in OA, where they are thought to have a protective effect on subchondral bone and cartilage. A recent meta-analysis failed to demonstrate symptomatic improvement or reduction in radiographic OA progression with bisphosphonate therapy (Table 1) [10]. However, reduced MRI BML size was demonstrated in two studies. It is likely that many of the studies assessing radiographic progression were underpowered and many did not use modern inclusion criteria that enable detection of a pain response (such as pain > 4- on a 10-point scale).

**Table 1:** Bisphosphonate intervention studies (adapted from [10])

Intervention vs comparator	Study duration	Number of participants	Main symptom outcome	Structural outcome	Summary of results
Risedronate 5 or 15 mg/day vs oral placebo [11]	12 months	284	WOMAC pain and function	X-Ray mean joint space width change (mm)	No significant difference between treatment groups
Risedronate 5 or 15 mg/day or 35 mg/week vs oral placebo [12]	24 months	1251	WOMAC pain and WOMAC function; patient global assessment	Proportion of patients experiencing X-Ray progression (defined as ≥ 0.6 mm of JSN over 24 months)	No significant difference between treatment groups
Risedronate 5 or 15 mg/day or 50 mg/week vs oral placebo [12]	24 months	1232	WOMAC pain and WOMAC function; patient global assessment	Proportion of patients experiencing X-Ray progression (defined as ≥ 0.6 mm of JSN over 24 months)	No significant difference between treatment groups
Zoledronic acid 5 mg vs IV placebo [13]	12 months	59	Visual analogue scale (VAS) pain	Change in total bone marrow lesion (BML) area on MRI	Significant VAS improvement in the per protocol analysis (no significant difference in intention to treat population) Significantly reduced BML size at 6 months but not 12 months
Clodronate 2 mg/week vs intra-articular placebo [14]	4 months	80	Sum of VAS pain on passive movement and digital pressure	N/A	Significant improvement at 2 months but not 4 months
Neridronate (100 mg 4 × over 10 days) vs IV placebo [15]	2 months	68	VAS pain	Change in BML size (semi-quantitative scoring)	Significant improvement in pain score and BML size

Recent studies have focused recruitment on patients with definite subchondral bone abnormalities, specifically MRI-detected bone marrow lesions (BMLs). BMLs are commonly seen on MRI scans of OA knees, represent areas of trabecular loss, microfracture and fibrosis, and have associations with both pain and progressive cartilage loss [16–19]. Zoledronic acid (ZA) was compared with placebo in a double-blind, parallel-group trial of 59 patients aged 50–80 with knee pain and at least one BML on MRI [13]. Results were promising, demonstrating a significant symptomatic benefit and reduction in BML size at 6 months. The preliminary report from the larger follow-up multicentre, randomised controlled trial has been recently presented. In this study, 223 knee OA patients with significant knee pain and MRI-detected BMLs received annual intravenous infusion of ZA 5 mg or placebo over 2 years [20]. Unfortunately, no significant improvement was detected at 24 months in the Western Ontario and McMaster Universities Osteoarthritis Index (WOMAC) pain scale, WOMAC function scale, or BML size change with ZA.

A sub-study of this 2-year ZA trial investigated the effects of ZA 5 mg plus intravenous methylprednisolone 10 mg in a preparation called VOLT01^17^. 117 patients with symptomatic knee OA were randomised to receive single dose VOLT01, ZA 5 mg monotherapy or placebo. The study’s primary outcome measurement was the incidence of the acute phase response following ZA administration (a flu-like or febrile response which can occur following IV bisphosphonate) and secondary outcomes measured BML size, WOMAC and visual analogue scale (VAS) pain and WOMAC function at 6 months. The incidence of acute phase responses was similar between treatment groups. In addition, VOLT01 and ZA were not superior to placebo in any of the secondary outcome pain measurements.

#### Summary

Recent studies have failed to demonstrate structural or symptomatic improvement with bisphosphonates in knee OA.

### Strontium Ranelate

Strontium ranelate has also been trialled in knee OA. The SEKOIA trial investigated strontium 1 or 2 g/day versus placebo in a randomised, double-blind, 36-month study involving 1683 patients with symptomatic primary knee OA [21]. A significant reduction in radiographic joint space width (JSW; a surrogate for hyaline cartilage loss) reduction with both strontium doses compared to placebo was demonstrated at 36 months. The results also demonstrated a significant improvement in WOMAC total score and pain subscore at 2 g/day, though the effect size was very small (approximately 0.1). However, with a 14% annualised dropout rate, participant retention in (much desired) long-term OA trials is difficult and handling consequent missing data remains a complex issue. Strontium is also contraindicated in cardiovascular disease—a co-morbid condition patients with OA are also at increased risk of developing [22].

#### Summary

Strontium may improve pain and structural progression in knee OA but the benefits are small, and given potential cardiovascular risk, its use as a treatment is unlikely.

### Sprifermin

Animal models of OA have demonstrated that fibroblast growth factor-18 can increase cartilage volume via proliferation of chondrocytes and modulating extracellular matrix turnover [23, 24]. Sprifermin is a recombinant human fibroblast growth factor-18 (rhFGF18) which is administered intra-articularly and targets FGFR3 receptors in cartilage [25]. Sprifermin was investigated in a phase I, 1-year, randomised, double‐blind, placebo‐controlled, proof‐of‐concept trial at doses of 10 μg, 30 μg, and 100 μg [26]. Subjects received 2 cycles of three once-weekly injections (at weeks 0–2 and 13–15) of sprifermin or placebo and the results from 168 patients were evaluated. Although sprifermin failed to achieve its primary efficacy endpoint of significant reduction in loss of central medial femorotibial compartment cartilage thickness at 6 or 12 months, the study did, however, achieve its pre-specified (and more sensitive) secondary structural efficacy MRI endpoints: significant reductions in loss of total femorotibial and lateral femorotibial cartilage thickness.

A further 5-year Phase II, dose-ranging, randomised trial of sprifermin (the FORWARD trial) is currently ongoing [27]. 549 patients were randomised to 1 of 5 groups: intra-articular injections of 100 microg of sprifermin administered 6-monthly (*n* = 110) or 12-monthly (*n* = 110); 30 microg of sprifermin 6-monthly (*n* = 111) or 12-monthly (*n* = 110); or placebo every 6 months (*n* = 108). Each treatment cycle consisted of 3 once-weekly injections and total treatment duration was 18 months. Structural endpoints (cartilage thickness) were measured by quantitative MRI at the tibiofemoral joint. There was an initial dose-dependent increase in overall cartilage thickness with sprifermin 100 μg for the first 2 years, though no significant improvement in WOMAC total score and pain, function, and stiffness subscale scores were detected between any of the treatment groups and placebo at 2 years [27].

Overall cartilage thickness reduced for all treatment groups between years 2 and 3 although the significant difference between sprifermin 100 μg 6-monthly and placebo groups was maintained [28]. The loss of cartilage during the period may reflect the cessation of treatment. Preliminary results at 3 years also demonstrated a reduction in the expected (natural history) mean cartilage thickness loss from baseline to 3 years in patients on Sprifermin 100 μg 6-monthly versus placebo at the total femorotibial joint, and in the medial, lateral, central medial and central-lateral sub-regions. 18.4% (sprifermin) and 24.1% (placebo) of participants discontinued the study within 3 years. At year 3 there was also no difference in symptom improvement between treatment groups, raising an important question regarding treatments improving OA structural pathologies: how long after structural change should patients be monitored in order to detect a difference in patient important outcomes (such as reduction in symptoms or joint replacement)?

The FORWARD study was primarily designed to assess structural progression and did not use modern pain trial inclusion criteria. Preliminary data from a post hoc analysis at year 3 of an ‘at-risk’ subgroup of patients with higher pain scores (WOMAC pain score of 40–90) and lower joint space width (1.5–3.5 mm) at baseline—which are both predictors of more responsive outcome measurement—reported that patients with reduced medial or lateral joint space width at baseline along with higher baseline pain scores had significantly greater WOMAC pain score improvements with the highest dose of sprifermin (100 μg 6-monthly) compared with placebo [29].

#### Summary

Sprifermin has demonstrated structural improvement in knee OA compared to placebo. Translation into symptomatic improvements is less certain and phase III trials are awaited.

### Wnt Pathway Inhibition

The Wnt signalling pathway is a signal transduction pathway implicated in cartilage breakdown and OA pathophysiology, through its effect on chondrocyte, osteoblast and synovial cell differentiation [30, 31]. Altered expression of genes encoding Wnt signalling pathway proteins have been demonstrated in murine and human OA tissues, along with lower levels of the Wnt inhibitory protein DKK1 [31]. Lorecivivint (formerly SM04690) is an inhibitor of the Wnt signalling pathway and also inhibits the enzymes CDC-like kinase 2 (CLK2) and dual-specificity tyrosine phosphorylation-regulated kinase 1A, enhancing chondrogenesis, chondrocyte function and reducing inflammation [32]. It has been investigated in a phase IIa randomised, double-blind, placebo-controlled trial comparing 3 doses (low, medium and high) in 455 patients with knee OA. No statistically significant improvement in WOMAC A1 pain (pain when walking on a flat surface) was demonstrated between treatment groups and placebo. Exploratory analyses investigated the hypothesis that patients with bilateral symptomatic knee OA would be less responsive to treatment than patients with only one knee affected (a ‘widespread pain’ interference phenomenon). Two pre-specified subpopulations were therefore defined: unilateral symptomatic knee OA (*n* = 164) and subjects with unilateral knee OA without widespread pain (*n* = 128). Medium dose (0.07 mg) SM04690 did significantly improve WOMAC A1 score versus placebo in unilateral symptomatic knee patients at 39 and 52 weeks and unilateral symptomatic patients without widespread pain at 26, 39 and 52 weeks [33]. Reduction in radiographic joint space width progression was also significantly greater in the 0.07 mg treatment group versus placebo at week 52, though patient numbers were small for structural assessment [34].

A further 24-week, phase IIb study randomised 695 patients to receive a single dose lorecivivint (0.03, 0.07, 0.15 or 0.23 mg), vehicle placebo or sham (dry needle only) at baseline. 635 subjects completed the study and the preliminary results demonstrated significant improvements from baseline in 0.07 mg and 0.23 mg lorecivivint dose groups compared to placebo for numeric rating scale pain (0.07 mg and 0.23 mg at weeks 12 and week 24), WOMAC Pain (0.07 mg at week 12; 0.23 mg at weeks 12 and 24), WOMAC Physical Function (0.07 mg at week 12; 0.23 mg at weeks 12 and 24) and patient global assessment (0.07 mg at week 12; 0.23 mg at weeks 12 and 24) [35]. In addition, a homogenous group of patients with joint space width 2–4 mm and no widespread pain demonstrated an improved symptom effect size vs placebo at 12 and 24 weeks compared to the full analysis set at 0.07 mg [36].

#### Summary

Sub-analyses of phase II trials suggest potential symptomatic and structural benefits with lorecivivint versus placebo. Await results of phase III trials which are underway.

### Cathepsin K Inhibition

Cathepsin K is a cysteine protease highly expressed in activated osteoclasts. It plays an important role in bone resorption, degrading types I and II collagen and aggrecan found in cartilage. MIV-711 is a novel potent, selective and reversible inhibitor of cathepsin K which inhibits the actions of osteoclasts and is associated with reduced expression of biomarkers of bone resorption and cartilage loss [37]. Cathepsin K inhibition has also recently been shown to reduce the risk of fracture in post-menopausal osteoporosis, although with an increased cardiovascular risk [38]. A multicentre, randomised, placebo-controlled, double-blind, three-arm parallel, Phase IIa study evaluated the efficacy, safety and tolerability of MIV-711 in patients with knee OA [39]. Patients received oral MIV-711 100 mg, 200 mg or matched placebo once daily for 26 weeks. No significant difference in pain reduction and quality of life scores were detected, although there was a trend towards increased reduction in pain scores with 100 mg and 200 mg doses at 26 weeks. There was a significant reduction in femoral OA bone disease progression on MRI at week 26 for MIV-711 100 mg and 200 mg doses versus placebo and significant reduction in loss of cartilage thickness on the medial femur for 100 mg dose versus placebo. A unique aspect of this trial was the key secondary outcome measure of 3D quantitative bone area using a machine learning method, enabling detection of change in a relatively small-number OA trial; this novel imaging biomarker can potentially be used as an outcome measure in further DMOAD trials [40]. The main adverse events reported were musculoskeletal symptoms, skin disorders and infections, although there was a reported overall acceptable safety profile [41]. Further studies are required to evaluate the potential structure-modifying effects of this agent.

#### Summary

Cathepsin K reduced knee OA structural progression but no significant symptom improvement over placebo was demonstrated over 6 months in a small phase II trial.

## Targeting Nerves

As might be expected from this these modes of action these therapies are symptomatic and not trying to modify joint structure.

### Intra-articular Capsaicin

Capsaicin is a chilli pepper extract which binds to a protein called transient receptor potential cation channel subfamily V member 1 (TRPV1), which is expressed on nociceptive nerve fibres (Aδ and C). It causes the burning sensation associated with chillies but is also considered an attractive target for potential analgesic medication, as its activation triggers a prolonged refractory state known as desensitisation [42]. CNTX-4975, the first intra-articular capsaicin preparation, is a highly purified, synthetic trans-capsaicin specifically targeting TRPV1-containing pain nociceptors [43]. Other sensory fibres such as touch or pressure are unaffected. Previous evidence supported topical capsaicin for relieving OA pain [44–47]. A phase II trial of single dose CNTX-4975 1 mg knee injection in patients with moderately painful knee OA was shown to improve the WOMAC A1 pain score (pain on walking) at weeks 12 and 24 in a 24-week, randomised, double-blind, placebo-controlled, dose-ranging study [48]. Data on adverse events are limited currently but the most common treatment-emergent adverse event is reported to be post-procedural pain, although this greatly subsides by 2 h post injection; in addition, there were no withdrawals due to side-effects [48]. Phase III trials of CNTX-4975 and a study examining the efficacy of repeated doses are currently in progress [49, 50].

#### Summary

Intra-articular capsaicin has demonstrated symptom improvement in knee OA. Phase III studies are in progress.

### Anti-nerve Growth Factor Monoclonal Antibodies

Nerve growth factor (NGF) is a neurotrophin that binds to TrkA and has increased expression in OA [51]. It stimulates the growth of nociceptive nerve fibres and expression of nociceptive cell surface receptors. Osteoarthritic knee joints are highly innervated with nerve fibres in the joint capsule, ligaments, periosteum, menisci, subchondral bone and synovium [52]. This makes the peripheral nociceptive pathway an attractive target for novel analgesic agents.

#### Tanezumab

Tanezumab, and fasinumab are monoclonal antibodies targeting NGF, preventing it from binding its receptor to reduce pain [53]. Tanezumab is a humanised IgG type 2 monoclonal antibody produced by Pfizer and Eli Lilly with high specificity for NGF. Most data are available on tanezumab and in a meta-analysis of 9 studies with 10 randomised controlled trials enrolling 7665 patients with knee or hip OA, intravenous tanezumab (2.5 mg, 5 mg and 10 mg) demonstrated superior efficacy compared to placebo/active comparator (significantly improving WOMAC pain subscale, WOMAC function subscale and patient global assessment) [54].

Fears of joint safety due to reported cases of rapidly progressive OA (RPOA) and then fears about possible sympathetic nerve problems (not borne out subsequently) meant this class of drugs underwent a number of regulatory holds, but are now back in development.

More recent studies have investigated subcutaneous (SC) preparations of tanezumab. A phase III trial of SC tanezumab compared fixed (2.5 mg) doses 8 weeks apart and step up dosing (2.5 mg administered at baseline, 5 mg administered at week 8) versus placebo in 696 OA hip/knee patients who had not responded to, or unable to tolerate, standard analgesia. Tanezumab 2.5 mg fixed and 2.5 mg/5 mg step up dosing was demonstrated to be superior to placebo in improving WOMAC Pain, WOMAC function and patient global assessment scores at week 16 [55]. There were more joint replacements observed in patients receiving tanezumab, although these were mostly elective and not associated with an AE. Two joint replacements were considered to be due to RPOA (see below).

Earlier phase III studies have demonstrated tanezumab monotherapy at 5 mg and 10 mg to have greater analgesic efficacy over NSAIDs (celecoxib 100 mg and naproxen 500 mg) and oxycodone 10–40 mg [56, 57]. Combination tanezumab and NSAID therapy also demonstrated significantly greater analgesic efficacy over NSAID monotherapy but not compared with tanezumab monotherapy [57]. A recent phase III study investigated SC tanezumab 2.5 mg or 5 mg versus NSAID (naproxen 500 mg, celecoxib 100 mg, or diclofenac ER 75 mg orally) in an 80-week study of 2996 patients with hip or knee OA. Tanezumab 5 mg significantly improved WOMAC pain and WOMAC function scores compared to the NSAID group at week 16 but not the Patient’s Global Assessment of OA. There were no significant improvements compared to NSAID with tanezumab 2.5 mg at 16 weeks or with either tanezumab doses at 56 weeks [58].

Tanezumab (as the most widely studied drug) has been associated with a number of adverse effects, although discontinuation rates in anti-NGF trials are low [54]. Reported side-effects include paraesthesia, headaches, arthralgia, peripheral oedema, peripheral neuropathy, hypo- and hyper- aesthesia. Lower doses of tanezumab are associated with fewer adverse events [54]. Arthralgia was the most commonly reported side effect (8% of tanezumab-treated patients).

#### Fasinumab

Fasinumab is also a humanised monoclonal antibody developed by Regeneron and Teva with high specificity and affinity for NGF. It was recently investigated in a phase IIb/III double‐blind, placebo‐controlled, randomised clinical trial of 421 patients with moderate-to-severe knee or hip OA and inadequate response or intolerance to analgesics [59]. Patients were randomised to receive fasinumab 1 mg, 3 mg, 6 mg, 9 mg or placebo every 4 weeks over 16 weeks with follow-up until week 36. 346 patients completed the study. Statistically and clinically significant improvements in WOMAC pain, WOMAC physical function subscale and patient global assessment scores were detected with all the doses of fasinumab compared to placebo at week 16. These improvements were not dose-dependent.

#### Rapidly progressive OA

Rapidly progressive OA (RPOA) is the most serious adverse event reported with the anti-NGF therapies, with the risk apparently dose-related [59, 60]. It is a painful condition diagnosed radiographically by rapid joint space narrowing and severe progressive atrophic bone [61]. Recent trials have used a maximal 5 mg dose of tanezumab as the RPOA risk is reduced and outweighed by its potential therapeutic benefit [62]. Although a higher rate of RPOA is observed with tanezumab 2.5 mg and 5 mg compared to NSAID [63], the combination of tanezumab and NSAIDs appears to increase the risk of RPOA compared to tanezumab alone [62], and modern trials have also been designed to reduce concomitant NSAID use.

#### Summary

Anti-NGF antibodies have demonstrated symptomatic improvement in hip and knee OA. Higher doses increase the risk of developing RPOA. These agents may be the first new OA therapy to emerge in many years, though careful evaluation of benefit-risk will be required.

## Immunomodulators

### Injectable Corticosteroids

Intra-articular corticosteroids have been shown to be effective in reducing OA pain, although their effects are short lived with no associated benefit seen at 6 months [64]. A Cochrane review of 27 trials investigating intra-articular corticosteroids in knee OA found an association with small to moderate improvement in function at up to 6 weeks, but no improvement beyond this timeframe [65]. Moderate to large heterogeneity between trials was also found.

#### Intra-articular Triamcinolone Acetonide Extended Release

Given the reported short-term benefits of intra-articular corticosteroid, FX006, a triamcinolone acetonide extended release (TA-ER) formulation produced using microsphere technology, with the aim of giving prolonged benefits, was investigated in patients with knee OA. TA-ER at doses 10 mg, 40 mg and 60 mg were compared in a phase IIa randomised, double-blind, controlled, dose-finding trial [66]. 228 patients with knee OA were followed up for 12 weeks post single intra-articular knee injection. There was a significant improvement in mean daily pain intensity scores (the primary outcome measure) with TA-ER 40 mg versus immediate-release triamcinolone 40 mg at weeks 5–10; furthermore, all WOMAC subscale scores were significantly improved with TA-ER 40 mg compared to immediate-release triamcinolone at 8 weeks. No superiority was demonstrated with TA-ER 10 mg and 60 mg versus immediate-release triamcinolone 40 mg in improving mean daily pain intensity, although TA-ER 10 mg was significantly superior to immediate-release triamcinolone 40 mg in improving some secondary efficacy endpoints. Similar frequencies of adverse events (AEs) were reported between TA-ER and immediate-release triamcinolone.

TA-ER was compared to placebo in a further phase IIB study of 306 knee OA patients. The primary outcome of a significant improvement in average daily pain (ADP) intensity versus placebo at 12 weeks was not achieved; however, TA-ER 32 mg significantly improved ADP intensity scores versus placebo at weeks 1–11 and at week 13 [67]. More recently, a phase III, multicentre, double-blinded, randomised, controlled trial comparing TA-ER 32 mg to immediate-release triamcinolone 40 mg and placebo in 484 knee OA patients achieved its primary endpoint of a significant improvement in ADP intensity compared to placebo at 12 weeks [68]. TA-ER did not significantly improve ADP intensity over immediate-release triamcinolone at 12 weeks; however, WOMAC pain, stiffness and physical function scores, and the Knee injury and Osteoarthritis Outcome Score (Quality of Life subdomain—KOOS-QOL) at 12 weeks were significantly improved with TA-ER 32 mg compared to both placebo and immediate-release triamcinolone. The differences with the active comparator seen when using different outcome measures suggests a greater responsiveness for the disease-specific, multi-item WOMAC tool over the ADP single item question. As TA-ER has been shown to be more effective than placebo, it has been licensed by the FDA for managing OA-related knee pain. A further advantage of TA-ER’s mechanism of action is reduced systemic exposure compared to immediate-release triamcinolone, due to its slower intra-articular release [69]. TA-ER 32 mg has been shown to have less effect on glycaemic control than standard triamcinolone 40 mg in type 2 diabetic patients [70].

#### Summary

Intramuscular and intra-articular steroid provides short-lived symptomatic benefit in OA. An extended release intra-articular steroid is now licensed in the USA.

### Hydroxychloroquine

Hydroxychloroquine is routinely used in the management of rheumatoid arthritis synovitis and has also been used in the management of inflammatory hand OA, related to anecdotal evidence of benefit and a tolerable safety profile [71, 72]. The immunomodulatory actions of hydroxychloroquine work through inhibitory effects on toll-like receptor (TLR) signalling [73]. This was considered potentially therapeutic in OA as TLRs are upregulated in OA cartilage tissue and trigger its breakdown via pro-inflammatory pathways [74, 75]. Furthermore, there is evidence of synovitis in hand OA [76, 77]. Early small pilot studies suggested symptomatic improvement following hydroxychloroquine treatment [78, 79]. A large randomised, double-blind, placebo-controlled clinical trial investigated this further in 248 patients over a 12-month period [80]. Patients with moderate-to-severe hand pain were randomised to hydroxychloroquine or placebo, along with their usual analgesic medication. No significant reduction in hand pain with additional hydroxychloroquine compared to placebo at 6 months was detected, therefore not achieving primary endpoint. Hydroxychloroquine also did not slow radiographic OA progression compared to placebo at 12 months. A subset of patients who were stratified for (commonly found) ultrasound-detected synovitis also did not demonstrate hydroxychloroquine efficacy. Another randomised controlled trial compared hydroxychloroquine 400 mg to placebo in 196 patients with hand OA (but not on concomitant NSAID or corticosteroid treatment) and did not detect a significant difference in pain scores after 24 weeks of treatment [81].

#### Summary

Latest evidence demonstrates hydroxychloroquine is ineffective in reducing patient symptoms in hand OA.

### Tumour Necrosis Factor Inhibitors

Evidence suggests that tumour necrosis factor alpha (TNF α) is implicated in OA pathogenesis [82]. However, studies of adalimumab have not shown it to be effective compared to placebo in reducing symptoms of hand OA [83, 84]. More recently, the HUMOR trial compared subcutaneous adalimumab 40 mg on alternative weeks versus placebo over 12 weeks in a crossover-design study of 43 patients with erosive hand OA and synovitis seen on magnetic resonance imaging (MRI) [85]. There was an 8-week washout period before treatment groups crossed over. Adalimumab did not significantly reduce visual analogue scale (VAS) scores compared to placebo. In addition, there were no significant differences detected for secondary outcome measures including change in MRI-detected synovitis and bone marrow lesions.

Another anti-TNF α agent, etanercept, was recently studied in a 1-year, double-blind, randomised, placebo-controlled, multicentre trial of 90 patients with symptomatic erosive inflammatory hand OA. The primary endpoint of a significant improvement in VAS pain at 24 weeks was not achieved with etanercept 50 mg weekly [86]. In addition, there was no significant treatment reduction in ultrasonographic or MRI-detected synovitis after 1 year. A significant reduction in MRI-detected bone marrow lesions in the interphalangeal joints of one hand was detected after 1 year with etanercept; however, this was in a very small subgroup (n = 10 in each treatment group).

#### Summary

Anti-TNF agents have not demonstrated symptomatic improvement versus placebo in OA.

### Interleukin-1 α and β Inhibition

Interleukin-1 alpha (IL-1α) and interleukin-1 beta (IL-1β) expression is increased within OA cartilage and synovial membrane [87, 88]. Raised IL-1 levels are also associated with increased expression of OA pathophysiology markers in affected fluids and tissue including catabolic enzymes, prostaglandins, nitric oxide and other markers [89]. Blockage of the interleukin-1 receptor has been shown to slow the progression of OA in animal models [90–93].

Anakinra, a recombinant form of interleukin-1 receptor antagonist (IL-1Ra), was investigated in a randomised, multicentre, double-blind, placebo-controlled study of 170 knee OA patients who received a single intra-articular injection of placebo, anakinra 50 mg, or anakinra 150 mg in their symptomatic knee. No significant difference in the mean WOMAC pain score improvements from baseline to week 4 could be detected between the treatment groups, although anakinra was well tolerated [94].

Lutikizumab (formerly ABT-981) is a novel human dual variabledomain immunoglobulin that targets and inhibits IL-1α and IL-1β [95]. This was investigated in the ILLUSTRATE-K trial, a randomised, double-blind, placebo-controlled, parallel-group phase II study comparing fortnightly subcutaneous injections of lutikizumab at 25 mg, 100 mg, or 200 mg in patients with knee OA over 50 weeks [96]. The study achieved its primary endpoint with preliminary results demonstrating a significant improvement in WOMAC pain score at 16 weeks with lutikizumab 100 mg compared to placebo. However, there was no significant improvement with lutikizumab 25 mg or 200 mg compared to placebo at this time point. Cartilage thickness, MRI synovitis, and other structural endpoints were similar between lutikizumab and placebo, although lutikizumab was generally well tolerated. With the lack of dose response and failure to meet structural endpoints, there is uncertainty regarding the clinical efficacy of lutikizumab in knee OA based on this study.

Lutikizumab was also trialled in patients with erosive hand OA, in a phase IIa, placebo-controlled, randomised study. Clinical and radiological outcomes were measured in 131 patients with hand OA as per ACR criteria (≥ 3 inflamed interphalangeal joints which are tender, swollen, or both, hand pain ≥ 6 (scale 0–10), and ≥ 1 erosive interphalangeal joint on X-ray). Subjects were given lutikizumab 200 mg (*n* = 67) or placebo (*n* = 64) every 2 weeks for 26 weeks. Preliminary data did not demonstrate a significant improvement in Australian/Canadian Hand OA Index (AUSCAN) pain scores with lutikizumab compared to placebo at 16 weeks. There was also no significant difference in X-Ray or MRI data between treatment groups [97].

However, there have been further developments in the use of IL-1 in treating OA, involving canakinumab, a monoclonal antibody targeting IL-1β. Previous in vitro studies of canakinumab on human chondrocytes demonstrated increased proteoglycan and reduced nitric oxide synthesis which may reduce cartilage breakdown [98]. Canakinumab was recently studied in the CANTOS trial: a very large randomised, placebo-controlled trial investigating the cardiovascular effect of subcutaneous canakinumab [99]. 10,061 patients with previous myocardial infarction and a high-sensitivity C-reactive protein level ≥ 2 mg/L on blood testing were given canakinumab doses of 50 mg, 150 mg or 300 mg every 3 months for a median of 3.7 years. The study demonstrated that canakinumab at a dose of 150 mg was associated with a significantly lower rate of recurrent cardiovascular events compared to placebo, independent of lipid-level lowering. Despite this being primarily a cardiovascular study, a post hoc study reported reduced incidence of OA symptoms (reported as adverse events) and total knee and hip replacements in the patients who received canakinumab [100]. The implications of these findings are unclear.

#### Summary

Although previous studies of IL-1 inhibition in OA did not demonstrate a symptomatic or structural benefit, recent analyses from the CANTOS study suggest benefits in reducing joint replacement; more research in this area is required.

### Methotrexate

Methotrexate has been investigated in a phase III trial of patients with knee OA, targeting the synovitis and associated pain in the condition [101]. 155 patients were randomised to receive methotrexate (dose escalating 10–25 mg over 8 weeks with maintenance dose at 25 mg or the highest tolerated dose) or placebo. Preliminary data demonstrated statistically significant improvements with methotrexate at 6 months for WOMAC stiffness and physical function, but not pain. This benefit was reduced at 12 months. As a secondary outcome measure, synovial volume was measured on MRI at baseline and 6 months with no significant difference between treatment groups.

Methotrexate has also been investigated in patients with erosive hand OA [102]. 64 patients were randomised to receive methotrexate 10 mg weekly or placebo. There was no significant difference between treatment groups in VAS pain score reduction at 3 months; therefore the study did not achieve its primary outcome. There was also no significant improvement in pain at 12 months; however, there appeared to be less structural progression in the methotrexate group, with more remodelling seen in erosive joints.

#### Summary

Usual dose methotrexate has demonstrated symptomatic improvement at 6 months in knee OAbut low dose has not been beneficial in hand OA. More consideration of methotrexate benefits are required as a potential analgesic therapy.

## Conclusion

Treatment options for osteoarthritis pain remain limited. Different joint structures have been targeted in pharmacological intervention studies of OA with variable levels of success. Despite structural improvement with a cartilage anabolic agent and an osteoclast inhibitor, their effect on symptoms are still under investigation. The limitations in developing novel therapeutics in OA are partly related to our limited understanding of the structure–pain relationship in OA, resulting in a current lack of attractive tissue target in OA. However, recent trial data of agents targeting peripheral nociceptive pathways in knee OA have been promising.
